# Genomic analysis of hypervirulent *Klebsiella pneumoniae* reveals potential genetic markers for differentiation from classical strains

**DOI:** 10.1038/s41598-022-17995-2

**Published:** 2022-08-11

**Authors:** Anton Spadar, João Perdigão, Susana Campino, Taane G. Clark

**Affiliations:** 1 Faculty of Infectious and Tropical Diseases, London School of Hygiene & Tropical Medicine, London, UK; 2 Research Institute for Medicines (iMed.ULisboa), Faculdade de Farmácia, Universidade de Lisboa, Lisboa, Portugal; 3 Faculty of Epidemiology and Population Health, London School of Hygiene and Tropical Medicine, Keppel Street, London, WC1E 7HT UK

**Keywords:** Microbial genetics, Bacterial genomics, Bacterial pathogenesis

## Abstract

The majority of *Klebsiella pneumoniae* (Kp) infections are nosocomial, but a growing number of community-acquired infections are caused by hypervirulent strains (hvKp) characterised by liver invasion and rapid metastasis. Unlike nosocomial Kp infections, hvKp are generally susceptible to antibiotics. Due to the rapid progression of hvKp infections, timely and accurate diagnosis is required for effective treatment. To identify potential drivers of the hypervirulent phenotype, we performed a genome-wide association study (GWAS) analysis on single nucleotide variants and accessory genome loci across 79 publicly available Kp isolates collected from patients’ liver and a diverse global Kp dataset (n = 646). The GWAS analysis revealed 29 putative genes (P < 10^–10^) associated with higher risk of liver phenotype, including hypervirulence linked salmochelin *iro *(odds ratio (OR): 29.8) and aerobactin *iuc* (OR: 14.1) loci. A minority of liver isolates (n = 15, 19%) had neither of these siderophores nor any other shared biomarker, suggesting possible unknown drivers of hypervirulence and an intrinsic ability of Kp to invade the liver. Despite identifying potential novel loci linked to a liver invasive Kp phenotype, our work highlights the need for large-scale studies involving more sequence types to identify further hypervirulence biomarkers to assist clinical decision making.

## Introduction

*Klebsiella pneumoniae* (Kp) is a Gram-negative pathogen increasingly capable of causing severe organ and life-threatening disease. Kp is classified across two main virulence phenotypes, classical (cKp) and hypervirulent (hvKp). CKp is the most common and normally a nosocomial infection, generally occurring among patients with additional co-morbidities^1^. Less common is hvKp, which is characterized by invasive infection within the community setting in otherwise healthy individuals, and with rapid metastatic spread. The typical hvKp presentation involves pyogenic liver abscesses, but also endophthalmitis, meningitis or necrotising fasciitis, all of which are unusual clinical manifestations for cKp. Epidemiologically, hvKp is more common in East and Southeast Asia but is an emerging threat in Europe, particularly when associated with carbapenemase producing clones^1–4^.

Biomarkers to differentiate cKp from hvKp are needed to inform diagnostic tests for application by clinical laboratories for optimal patient care and for use in epidemiological surveillance and research studies. However, a complete set of robust biomarkers is not available. Several genetic loci have been identified as virulence factors in Kp, primarily using murine models of infection. These include gene clusters associated with the synthesis of accessory siderophore systems yersiniabactin (*ybt*, *irp1*, *irp2*, and *fyuA*), aerobactin (*iucABCD*, *iutA*), colibactin (*clbA-R*), salmochelin (*iroN*, *iroBCD*), or microcin; mucoidy phenotype regulators (*rmpA* and *rmpA2*), which can up-regulate capsule production; an allantoinase gene cluster; the ferric uptake operon *kfuABC*; and the two-component regulator *kvgAS*, and the K1, K2 and K5 capsular serotypes^1,5–8^. The combination of salmochelin, aerobactin, and *rmpA* is frequently, but not always, linked to the presence of genes from the known Kp virulence plasmids such as pLVPK and pK2044. Some of these may be correlated with hypervirulence^5^, but results are inconsistent. In a study of Kp samples from liver abscess samples in East China, only 29% of samples were of hypermucoviscous phenotype^9^. Similarly, while the accessory salmochelin locus is frequently found in hvKp samples^6^, experimental evidence indicates that aerobactin is the main driver of hypervirulence^10^.

Here we analysed the core genome and shared accessory genes of all publicly available Kp samples sourced from the liver (n = 79) and compared them to a large globally diverse public Kp dataset (n = 646) using robust statistical association and cluster analysis methods. Unlike previous studies which leveraged in vivo models in either mice (*Mus musculus)* or moths (*Galleria mellonella*) to determine hypervirulence^7,8^, we looked at isolates collected from patients’ liver, which is a typical clinical presentation site of hvKp. We have found that both accessory *iro* and *iuc* loci are strongly associated with liver isolates, and the hypermucoidy associated gene *rmpA* was not linked to hypervirulence. Whilst the analysis revealed new putative loci for the risk of liver phenotype, a minority (19%) of liver isolates did not have any of these markers. Although, the liver phenotype may be subject to misclassification, Kp may have intrinsic ability to colonise the organ, and its genetic underpinning will require a large-scale study to uncover the full repertoire of hypervirulence genes.

## Results

### Dataset characteristics

We analysed 79 hvKp isolates defined as samples isolated from patients’ liver. These were collected in China (n = 39), Singapore (n = 26), USA (n = 8), Brazil (n = 2) and one sample each from Ecuador, Guadeloupe, South Korea, and Viet Nam (Table 1). Of the 36 sequence types (STs) present in the 79 hvKp samples, ST23 was the most frequent (n = 27) followed by ST86 (n = 9) and ST258 (n = 4). All other STs had two or fewer samples. The 79 hvKp were compared to a large dataset of Kp isolates. This large dataset consisted of two groups: (i) 520 Kp assemblies with similar locations and collection dates to liver isolates, representing the broader genetic landscape of the bacterium; (ii) 126 Kp isolates from three hospitals in Thailand^11^, used to assess if our analytical approach was robust, especially to overfitting during data dimensional reduction. Overall, the resulting comparison dataset (n = 646) had samples from 302 different STs among which ST23 (n = 17), ST15 (n = 29), ST147 (n = 29), ST11 (n = 25) were the most common.

**Table 1:** Characteristics of study samples.

Characteristic	Liver samples (n = 79)		Non-liver samples (n = 646)	
	N	%	N	%
**Sequence types**				
ST23	27	34	17	3
ST86	8	10	4	1
ST258	4	5	13	2
ST15	–	–	29	4
ST147	–	–	29	4
ST11	1	–	25	4
Other	39	53	529	81
**Region**				
China	39	49	51	8
Singapore	26	33	1	0
USA	8	10	84	13
South America	4	5	10	2
South Korea	1	1	3	0
Viet Nam	1	1	–	–
Other	0	–	497	77
**O types**				
O1v1	22	28	147	23
O1v2	39	49	111	17
O2	8	10	152	24
O3	5	6	111	17
Other	5	6	125	19
**Carbapenemases**				
None	74	94	476	74
KPC-2	4	5	50	8
KPC-3	1	1	25	4
NDM-1	–	–	26	4
Other	–	–	69	11
**Aerobactin**				
iuc1	45	57	56	9
iuc2	5	6	1	0
iuc3	3	4	11	2
Other	–	–	8	1
None	26	33	570	88
**Salmochelin**				
iro 1	42	53	43	7
iro 1; iro 3	2	2	1	0
iro 2	5	6	2	0
iro 3	10	13	5	1
Other*	2	2	3	0
None	18	22	592	92

Sequence types (ST); O-types, carbapenemases and siderophore genotypes were determined by Kleborate software; *not reported by Kleborate software.

### Association analysis of liver invasive phenotype

**Figure 1:**
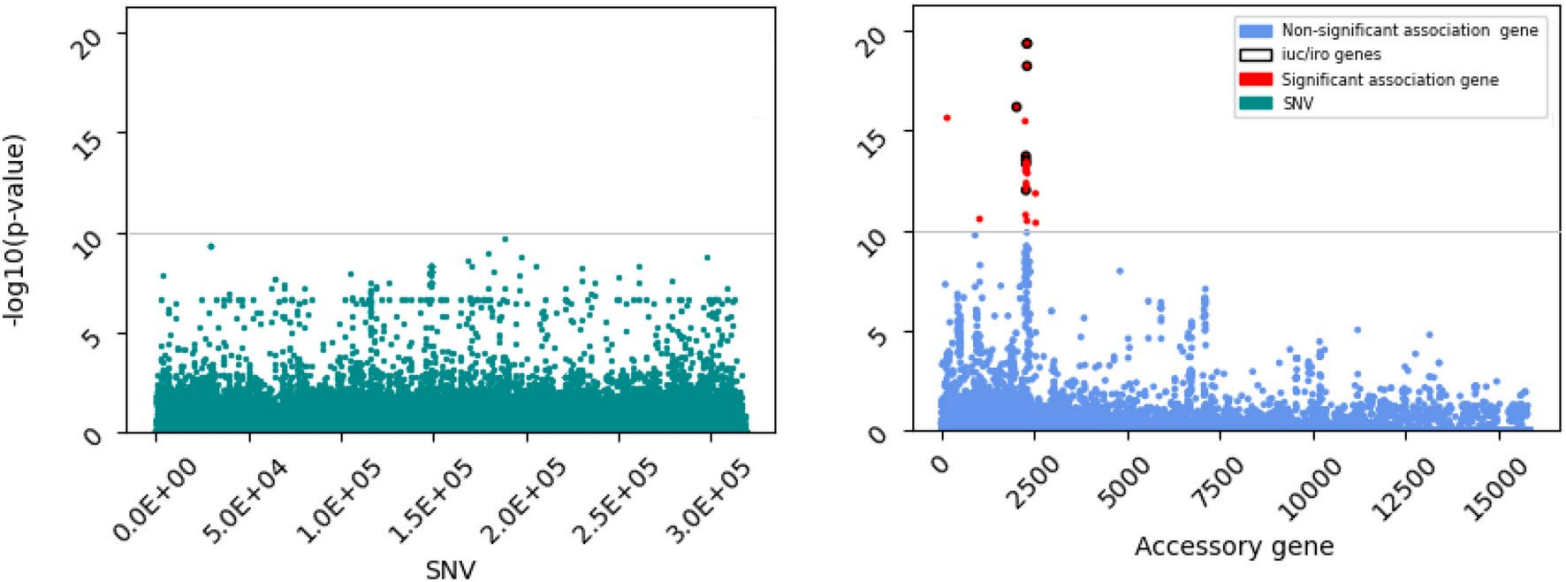
Association analysis of liver versus non-liver against individual genome-wide SNVs (n = 318,458) in the core genome (**A**) and accessory genes (n = 15,852) (**B**), accounting for population structure. Each point represents a result from single SNV or gene, and P < 10^–10^ is the significance threshold.

**Figure 2:**
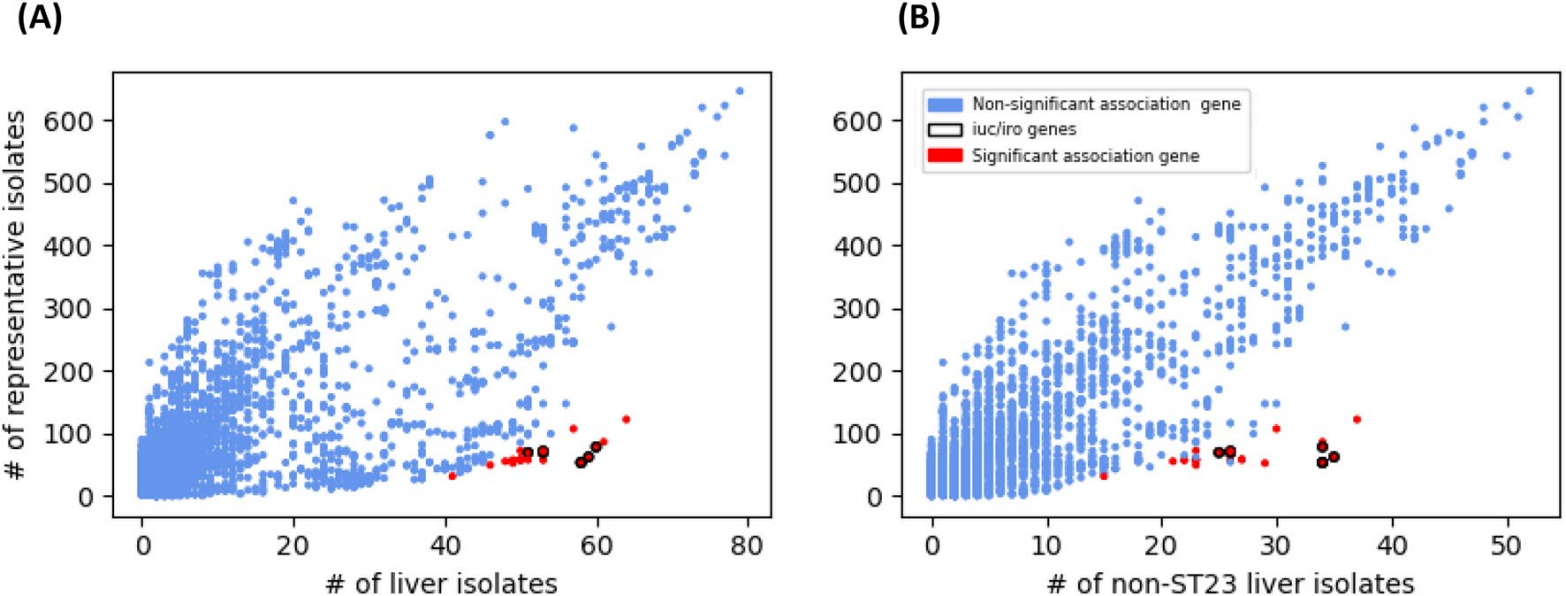
Frequency of accessory genome genes in all liver (**A**) (n = 79) and non-ST23 (**B**) (n = 52) liver isolates versus representative dataset (n = 646). The *iro* and *iuc* outliers are clearly visible. Each point is a gene, and the legend is consistent with Fig. 1.

We identified single nucleotide variants (SNVs) in the core genome (5.4 Mbp; 318,458 SNVs, with minor allele frequency (MAF) of 3 Kp isolates). We used a genome-wide association study (GWAS) strategy to identify any SNVs associated with the liver invasive phenotype, adjusting for population structure (Fig. 1A). None of the SNVs associations met our stringent statistical significance level (P < 10^–10^). A similar gene-wide analysis was performed on the presence or absence of accessory loci (n = 15,852), determined from robust assembly of contigs. Whilst the frequency of accessory genes in representative and liver isolates is the broadly correlated (rho = 0.79), the overrepresentation of ST23 (34%) among liver isolates leads to non-linearity (Fig. 2A), which improves when ST23 liver isolates are removed (Fig. 2B) (rho = 0.89). The clustering of isolates based on accessory genome demonstrates that the related genes are linked to ST and not geography, with ST23 being a tight cluster (Fig. S1). We performed the GWAS analysis accounting for this clustering, and found 29 putative genes associated with higher risk of liver phenotype, including known hypervirulence loci *iro* (odds ratio (OR): 29.8) and *iuc* (OR: 14.1), three further metal transport related genes, c-type lysozyme inhibitor (OR: 14.5) and 8 unannotated loci that could not be annotated (P < 10^–10^; Fig. 1B; Table 2). These accessory loci are of lower frequency in representative samples compared to liver isolates, irrespective of inclusion of ST23 (Fig. 2). Of the 79 liver isolates, 15 (19.0%) had none of these 29 putative accessory genes associated with liver invasive phenotype.

**Table 2:** Relative abundance of accessory genes associated with liver invasive phenotypes identified in Fig. 1B.

GeneID	Description	No. of times gene occurs in isolates			Association	
		Liver non-ST23 (n = 52)	Liver ST23 (n = 27)	Non-liver (n = 646)	Odds ratio	− log10 P-value
B385452	iroC	34	24	53	29.84	19.33
B385338	iroD	34	24	53	29.84	19.33
B603951	Siderophore enterobactin receptor FepA	35	24	62	23.64	18.19
B362201	iroB	34	26	78	24.68	16.32
B58052	EamA family transporter (*peg-344*)	34	27	86	22.76	15.58
B538146	IS21 family transposase	26	27	57	19.67	15.44
B381713	iucA	26	27	69	14.96	13.48
B385021	rmpC	29	20	52	13.04	13.37
B381836	iucB	26	27	70	14.40	13.28
B597737	Class I SAM-dependent methyltransferase	37	27	122	15.53	13.13
B382206	Ferric aerobactin receptor IutA	26	27	71	14.12	13.11
B382081	iucD	26	27	71	14.12	13.11
B381588	MFS transporter	26	27	71	14.12	13.11
B382762	DM13 domain-containing protein	23	27	57	15.15	13.02
B382654	Hypothetical protein	23	27	57	15.15	13.02
B382870	Hypothetical protein	23	27	57	15.15	13.02
B381162	c-Type lysozyme inhibitor	23	27	58	14.54	12.80
B382331	Hypothetical protein	23	27	58	14.54	12.80
B381271	Peptide deformylase	23	27	58	14.54	12.80
B385565	Hypothetical protein	27	24	58	13.35	12.65
B385675	Hypothetical protein	27	24	58	13.35	12.65
B382547	Hypothetical protein	22	27	57	13.83	12.18
B382440	TetR/AcrR family transcriptional regulator	22	27	57	12.96	12.04
B381960	IucA/IucC family siderophore biosynthesis protein	25	26	69	11.83	11.79
B402327	Tn3 family transposase	21	27	55	12.58	11.67
B380773	Alpha/beta hydrolase	23	27	72	9.90	10.61
B239784	Hypothetical protein	30	27	107	8.24	10.38
B385127	Putative protein	23	23	49	10.78	10.35
B402432	Hypothetical protein	15	26	31	13.66	10.26

The DNA sequences for each gene are in Data S2.

### Association between identified biomarkers and the rest of the accessory genome

**Figure 3:**
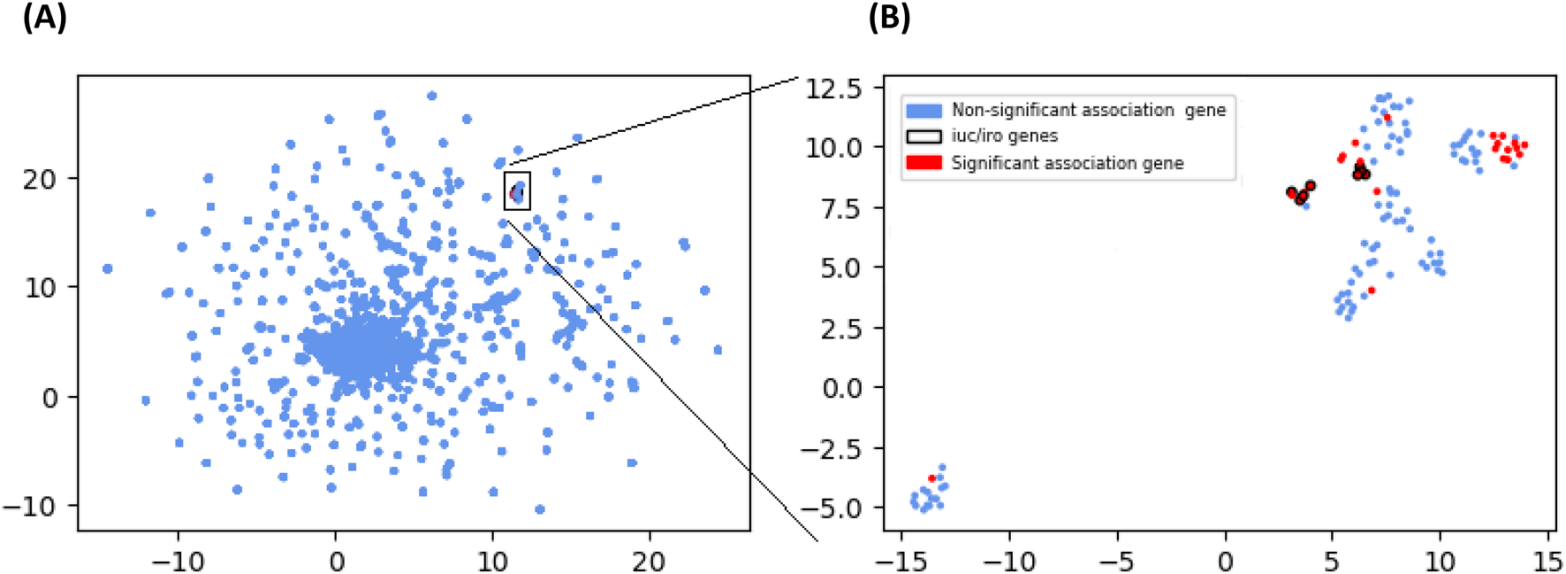
Cluster analysis of accessory genes. (**A**) Projection of genes presence/absence matrix into a *umap* 2-dmiensional view; (**B**) Structure of the *iro* and *iuc* containing gene cluster in (**A**). The liver phenotype genes (Table 2) are visible both in (**A**) and in greater detail in (**B**) for which the dimensional reduction algorithm was re-ran with subset of genes in (**A**). The axes are dimensionless. Each point is an accessory gene.

Having identified 29 accessory genes, including *iro* and *iuc*, with strong potential associations with the hvKp phenotype, we were interested in how they relate to each other i.e., their co-existence. As summarised in a recent review^12^, plasmids such as pLVPK, pK2044 and pSGH10 are known carriers of hypervirulence associated genes. Because identified biomarkers do not occur at the same frequency, we hypothesised that they may be on different parts of the hypervirulence plasmids. To test this hypothesis, we performed a cluster analysis of all accessory genes using a *umap* (principal component-like) approach (see “Materials and methods”) (Fig. 3). All 29 association loci fell within a cluster of 121 (92 additional) genes (Fig. 3A; Data S3). By focusing on this cluster, *iro* and *iuc* loci are parts of different gene groups (Fig. 3B) consistent with these loci occurring independently of each other, and potentially linked to different hypervirulence plasmids (Fig. S2).

### Association between liver invasive phenotype and plasmid replicons

We evaluated the prevalence of the plasmids identified. Using PlasmidFinder nomenclature, pLVPK, pK2044 and pSGH10 carry IncHI1B(pNDM-MAR) replicons. In pLVPK and pK2044 the replicon sequences are identical. However, based on visual examination of sequences, the first 97nt of pSGH10 are different, while the remaining 472nt are identical to pLVPK and pK2044. In our dataset, 100 isolates had a pLVPK/pK2044 type sequence (20/100; 20.0% liver isolates), while 39 isolates had a pSGH10 type replicon sequence (24/39; 61.5% liver isolates) (Table 3). We observed that pSHG10 type replicons occurred almost exclusively in ST23 isolates (37/39), while a pLVPK/pK2044 type was much more widely distributed, with ST86 (11/100) being most frequent. There was a further variant of IncHI1B(pNDM-MAR) present in single liver isolates from South Korea, which differed from the above variants in the first 120nts. Overall, the most frequent replicon family among liver isolates was IncHI1B(pNDM-MAR) (45/79) followed by IncFIB(K) (16/79).

**Table 3:** Prevalence of IncHI1B(pNDM-MAR) plasmid replicons.

Replicons	Total	STs (no. isolates)	Countries (no. isolates)	From liver	With iuc	With iro
IncHI1B(pNDM-MAR) [pLVPK/pK2044 type]	100	ST86 (11), ST23 (6), ST15 (6), ST14 (5)	China (26), Thailand (24), Singapore (8), USA (5), United Kingdom (4)	20 (20.0%)	48 (48.0%)	39 (39.0%)
IncHI1B(pNDM-MAR) [pSHG10 type]	39	ST23 (37), ST1941 (1), ST152 (1)	China (15), Singapore (12), Thailand (8)	24 (61.5%)	39 (100%)	36 (92.3%)

### Liver isolates without identified biomarkers

Fifteen (19.0%) of the 79 liver Kp isolates did not have the 29 accessory genes associated with the liver phenotype, and included four ST258, two ST1165 and 9 other sequence types. Assuming that the liver invasive phenotype was not misclassified for these 15 samples, we investigated whether there were any other genes in the accessory genome that differentiated this group from the representative set. By examining differences in allele frequencies between the 15 isolates versus the representative set, we did not find any plausible biomarkers (Figure S3A). We also repeated the core genome GWAS for these 15 samples, but once again there was no SNV which reached the significance cut-off (all P > 10^–10^). It is possible that a combination of accessory genes can predict the phenotype, and we employed nine different machine learning approaches to assess if such a complex gene relationship exists. The imbalance between the 15 hvKp and 646 representative isolates can lead to poor classifier performance in machine learning models, so we ran 100 different datasets with the 15 liver and 15 randomly chosen representative isolates. The resulting predictive accuracy across all approaches was no better than 50% of the random guess (Figure S3B), suggestive that there are no strong predictors of the 19% of liver isolates in our dataset.

## Discussion

Hypervirulent Kp (hvKp) infections are an emerging global threat with biomarkers needed to differentiate underlying isolates from classical Kp, thereby informing clinical decision making. Previous genetic investigations for hvKp biomarkers have relied on animal models^7,8^, where in vivo work has identified and focused on both salmochelin *iro* and aerobactin *iuc* loci, sometimes together with genes also present on virulence plasmids. Experimental work has demonstrated that aerobactin is important for Kp survival and growth in human ascites and serum^10^. Additionally, in chicken *E. coli* infection models, both aerobactin and salmochelin have been shown to enhance the colonisation potential of Kp^13,14^. In contrast, our *in-silico* analysis explored 79 Kp samples isolated from the liver, where a liver invasion phenotype is a strong indicator of hvKp. By comparing these isolates with a broader large Kp dataset (n = 646) using a GWAS approach, we found biomarkers on the accessory genome associated with liver hvKp. These markers included *iro* [B] and *iuc* [ABD] loci, as well as *fepA* (a siderophore enterobactin receptor), *IutA* (a ferric aerobactin receptor), *IucA/IucC* (siderophore biosynthesis proteins), and several hypothetical proteins, which serve as candidates for future experiments. *RmpA*, which confers a mucoid phenotype was not found to be associated at our stringent statistical cut-off (P < 10^–10^), but these findings are consistent with recent work in carbapenem-resistant Kp^15^. Further, *rmpC* was identified in our GWAS, and ΔrmpC has been shown to maintain the downregulated expression of capsule genes but preserve hypermucoviscosity^16^ Another interesting gene is putative c-type lysozyme inhibitor that appears linked to the *iuc* [ABCD] locus. The presence of this gene is potentially associated with the typical clinical manifestation of hvKp in liver and eyes, both organs with high levels of lysozymes^17^.

Whilst most of the liver phenotype could be explained through accessory genes, a minority set of isolates did not have any apparent biomarkers. This observation may be explained by phenotypic misclassification where meta data is incorrect, the liver invasive phenotype being intrinsic to Kp, or due to rarely observed genes. Whilst Kp isolate sequence data are likely to be sourced from patients’ liver samples, the use of an in vivo hypervirulence phenotype can assist phenotypic-genotypic analysis. It is also possible that isolates with known *iuc* and *iro* markers are more likely to be reported compared to samples with undetermined virulence factors. To assess for the presence of sample selection bias, we included a large geographically concentrated dataset from Thai hospitals^11^, and consequently found it was not an outlying population in combined analyses with the diverse large global collection. Another limitation is the small number of available hvKp sequences and overrepresentation of the ST23 sequence types. Although, our work is one of the largest hvKp genomic investigations to date, there is a need for larger studies to close knowledge gaps in hvKp epidemiology, pathogenesis, host susceptibility, optimal treatment, and appropriate infection control measures.

Overall, with the increasing prevalence of hvKp strains globally, robust biomarkers of related infection are needed. Our GWAS approach has identified known and novel accessory loci associated with the liver invasive phenotype, some requiring experimental follow-up. It is possible that Kp has an intrinsic ability to invade the liver, requiring larger scale studies to understand the full repertoire of genes underlying hvKp, and thereby improve clinical decision making.

## Materials and methods

### Dataset

We identified potential hvKp samples with sequencing data by searching the NCBI Isolates Browser^18^ (November 2021) using key words “liver” and “hepa”. Metadata of positive hits were manually examined to confirm a likely liver invasive phenotype. We did not identify any samples isolated from endophthalmitis, which is an infrequent manifestation of hvKp. The search resulted in 79 samples, of which 31 had sequencing reads and 48 were sequence assemblies. We assembled the sequencing reads for all samples using Unicycler v0.4.8^19^ with a quality check performed using Busco software (v4)^20^ to ensure > 95% completeness and < 5% fragmentation of genes in the gammaproteobacteria_odb10 gene set. For consistency of downstream analysis, all samples were re-annotated with prokka software (v1.14.6)^21^ using the Klebsiella genus database^22^ and default settings.

The 79 hvKp samples were complemented by 520 randomly selected assemblies also from the NCBI Isolates Browser. However, before the random selection we identified groups of isolates matching by location, isolation source and create date. We removed all but one representative isolate from each group, to minimize bias from large, localized studies. These randomly chosen samples may have characteristics of hvKp, but they provide an important comparison for establishing if a set of genes is more common in hvKp compared to those in the broader population. We also enriched our dataset with a further 126 samples^11^ isolated from three hospitals in Thailand, to evaluate the impact of samples chosen from a small geographic area with a diversity of STs and assess the robustness of analysis. If our methods are prone to generating bias, we would expect this dataset to stand out, but it did not (see Fig. S1). The comparison dataset of 646 isolates consisted of 302 different STs with ST15 (n = 29), ST147 (n = 29), ST11 (n = 25) being the most common. Kleborate software (v2.1.0)^23^ was used to profile the isolates’ virulence and ST (Data S1).

### Analysis

The genes from all assemblies were clustered in a reference independent manner. The Kp core genome was identified as those genes which are not accessory. To identify a core genome, BLASTn (v2.9.0)^24^ with word-size 20 was used to find and remove genes that shared > 90% identity, were within 20% of median length of all such genes, and were present in > 90% of samples. A sensitivity analysis performed with alternative parameters produced similar results. This approach identified a conserved core gene set which was removed. For the remaining genes we performed an all versus all BLASTn search with word-size 11. We assigned genes to groups based on > 60% identity between any two genes intra group and < 20% length difference from median gene length intra group. The input for subsequent analysis was a 15,852 × 725 matrix with rows as gene groups and columns as samples, where individual cells are a binary value with one indicating that sample contains a gene from the group, zero otherwise. Genes were aligned using MAFFT software (v7.467)^25^ and the resulting alignment files transformed into a 318,458 × 725 python matrix, where rows are individual SNVs and columns are isolates.

Logistic regression models were used to find associations between the liver phenotype and SNVs or presence of accessory genes. These models included principal components for the population structure, and were implemented using statsmodels software (v0.13.0)^26^. The projection of the dataset into two dimensions was performed using the umap library (v0.5.1)^27^ in python using “hamming” distance. Clusters were determined using DBSCAN^28^ as implemented in sklearn (v0.24.2)^29^. Machine learning analysis was performed using sklearn functions to identify predictors of the liver phenotype. Plasmid replicons were identified using PlasmidFinder software (v2.1.1) with default settings^30^. The scripts for accessory genome construction are available at https://github.com/AntonS-bio/accessoryGenomeBuilder. The analysis scripts are available at https://github.com/AntonS-bio/KpHypervirulence.

### Ethics approval and consent

No ethics approvals were required as all data is publicly available.

## Supplementary Information

Supplementary Figures.

Supplementary Information 1.

Supplementary Information 2.

Supplementary Information 3.

## Data Availability

All data used in this work is publicly available in NCBI database (https://www.ncbi.nlm.nih.gov/). A list of isolates is in Data S1. Analysis scripts are available at https://github.com/AntonS-bio.
