# Supplementary material for: Genomic analysis of hypervirulent Klebsiella pneumoniae reveals potential genetic markers for differentiation from classical strains
Source: Sci Rep. 2022 Aug 11;12:13671. doi: 10.1038/s41598-022-17995-2 (PMC9372168; doi:10.1038/s41598-022-17995-2)
Supplement: Supplementary file 1 — Supplementary Figures. [file 41598_2022_17995_MOESM1_ESM.docx]

**Supplementary Information**

**Figure S1.** Clustering of isolates based on accessory genome demonstrates that accessory genes are linked to sequence type (ST) and not geography with ST23 being a tight cluster. Each point is an isolate and axis are dimensionless.


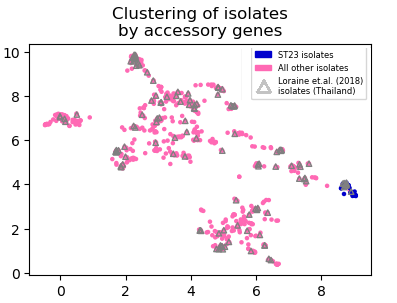


**Figure S2**. **(A)** This figure is the same as **Figure S1A**, except genes are coloured by clusters identified by DBSCAN algorithm. **(B)** The location of clusters of genes in **(A)** on a *K. pneumoniae* virulence plasmid pLVPK. Genes from same clusters occur together on the plasmid, but sometimes they are split-up by genes from another cluster.

**(A) (B)**


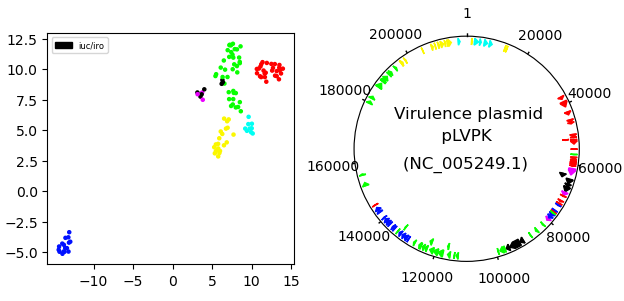


**Figure S3**. **(A)** Frequency of accessory genome genes in liver samples lacking *iro* and *iuc* loci versus representative dataset. **(B)** Phenotype prediction accuracy of different machine learning algorithms based on 100 iterations per algorithm. Each iteration was based on all 15 liver isolates lacking *iro* and *iuc* as well as 15 isolates randomly chosen from representative dataset.

**(A)** **(B)**


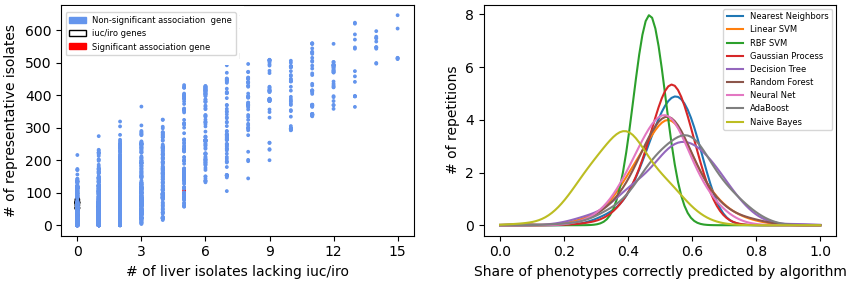


**Data S1**. Summary of isolates used in the study (Text file)

**Data S2**. Fasta sequences for all genes in **Figure 3B** and **Data S3**

**Data S3**. List of all genes in **Figure 3B**
